# Epidemic curves made easy using the R package *incidence*

**DOI:** 10.12688/f1000research.18002.1

**Published:** 2019-01-31

**Authors:** Zhian N. Kamvar, Jun Cai, Juliet R.C. Pulliam, Jakob Schumacher, Thibaut Jombart

**Affiliations:** 1 MRC Centre for Outbreak Analysis and Modelling, Department of Infectious Disease Epidemiology , School of Public Health, Imperial College London, London, UK; 2 Ministry of Education Key Laboratory for Earth System Modelling, Department of Earth System Science, Tsinghua University, Beijing, 100084, China; 3 South African DST-NRF Centre of Excellence in Epidemiological Modelling and Analysis (SACEMA),, Stellenbosch University, Stellenbosch, South Africa; 4 Gesundheitsamt Reinickendorf, Berlin, Germany; 5 Department of Infectious Disease Epidemiology, London School of Hygiene & Tropical Medicine, London, UK

**Keywords:** epicurve, incidence, epidemics, outbreaks, R

## Abstract

The epidemiological curve (epicurve) is one of the simplest yet most useful tools used by field epidemiologists, modellers, and decision makers for assessing the dynamics of infectious disease epidemics. Here, we present the free, open-source package incidence for the R programming language, which allows users to easily compute, handle, and visualise epicurves from unaggregated linelist data. This package was built in accordance with the development guidelines of the R Epidemics Consortium (RECON), which aim to ensure robustness and reliability through extensive automated testing, documentation, and good coding practices. As such, it fills an important gap in the toolbox for outbreak analytics using the R software, and provides a solid building block for further developments in infectious disease modelling. *incidence* is available from https://www.repidemicsconsortium.org/incidence.

## Introduction

Responses to infectious disease epidemics use a growing body of data sources to inform decision making ( Cori *et al*., 2017; Fraser *et al*., 2009; WHO Ebola Response Team *et al.*, 2014; WHO Ebola Response Team *et al*., 2015). While new data—such as whole genome pathogen sequences—are increasingly useful complements to epidemiological data ( Gire *et al*., 2014), epidemic curves—which describe the number of new cases through time (incidence)—remain the most important source of information, particularly early in an outbreak. Specifically epidemic curves(often referred to as ‘epicurves’) represent the number of new cases per time unit based on the date or time of symptom onset.

While conceptually simple, epicurves are useful in many respects. They provide a simple, visual outline of epidemic dynamics, which can be used for assessing the growth or decline of an outbreak ( Barrett *et al*., 2016; Fitzgerald *et al*., 2014; Jernberg *et al*., 2015; Lanini *et al*., 2014; Nhan *et al*., 2018) and therefore informing intervention measures ( Meltzer *et al*., 2014; WHO Ebola Response Team *et al.*, 2014; WHO Ebola Response Team *et al*., 2015). In addition, epicurves also form the raw material used by a range of modelling techniques for short-term forecasting ( Cori *et al*., 2013; Funk *et al*., 2018; Nouvellet *et al*., 2018; Viboud *et al*., 2018) as well as in outbreak detection algorithms from syndromic surveillance data ( Farrington & Andrews, 2003; Unkel *et al*., 2012).

Because of the increasing need to analyse various types of epidemiological data in a single environment using free, transparent and reproducible procedures, the R software ( R Core Team, 2017) has been proposed as a platform of choice for epidemic analysis ( Jombart *et al*., 2014). But despite the existence of packages dedicated to time series analysis ( Shumway & Stoffer, 2010) as well as surveillance data ( Höhle, 2007), a lightweight and *well-tested* package solely dedicated to building, handling and plotting epidemic curves directly from linelist data (e.g. a spreadsheet where each row represents an individual case) is still lacking.

Here, we introduce *incidence*, an R package developed as part of the toolbox for epidemics analysis of the R Epidemics Consortium ( RECON) which aims to fill this gap. In this paper, we outline the package’s design and illustrate its functionalities using a reproducible worked example.

## Methods

### Package overview

The philosophy underpinning the development of *incidence* is to ‘do the basics well’. The objective of this package is to provide simple, user-friendly and robust tools for computing, manipulating, and plotting epidemic curves, with some additional facilities for basic models of incidence over time.

**Figure 1.**
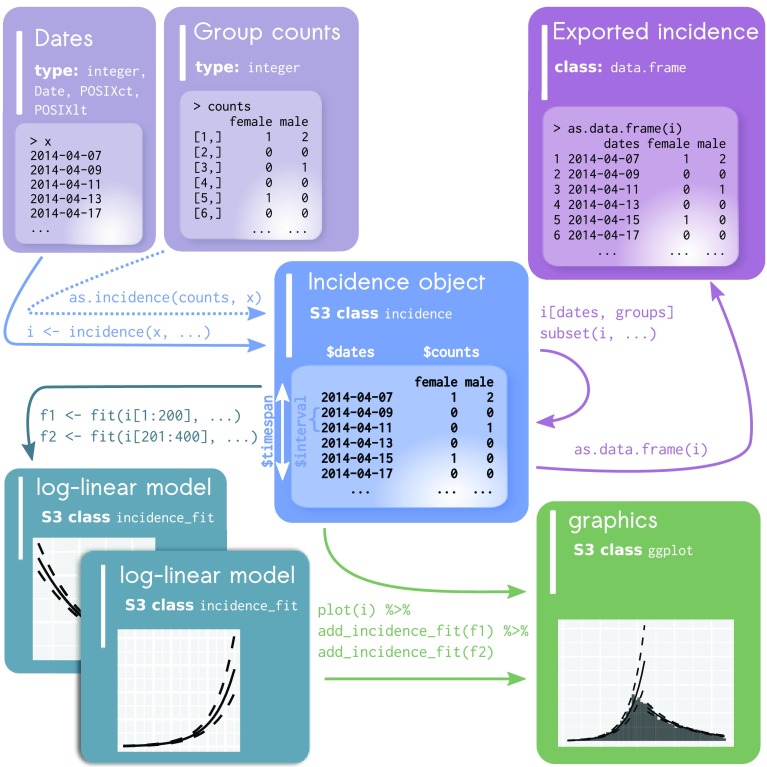
Generalized workflow from incidence object construction to modeling and visualization. The raw data is depicted in the top left as either a vector of dates for each individual case (typical usage) or a combination of both dates and a matrix of group counts. The incidence object is created from these where it checks and validates the timespan and interval between dates. Data subsetting and export is depicted in the upper right. Data visualization is depicted in the lower right. Addition of log-linear models is depicted in the lower left.

The general workflow ( Figure 1) revolves around a single type of object, formalised as the S3 class **incidence**. **incidence** objects are lists storing separately a matrix of case counts (with dates in rows and groups in columns), dates used as breaks, the time interval used, and an indication of whether incidence is cumulative or not ( Figure 1). The **incidence** object is obtained by running the function incidence() specifying two inputs: a vector of dates (representing onset of individual cases) and an interval specification. The dates can be any type of input representing dates including **Date** and **POSIXct** objects, as well as numeric and integer values. The dates are aggregated into counts based on the user-defined interval representing the number of days for each bin. The interval can also be defined as a text string of either "week", "month", "quarter", or "year" to represent intervals that can not be defined by a fixed number of days. For these higher-level intervals, an extra parameter— standard—is available to specify if the interval should start at the standard beginning of the interval (e.g. weeks start on Monday and months start at the first of the month). incidence() also accepts a groups argument which can be used to obtain stratified incidence. The basic elements of the **incidence** object can be obtained by the accessors get_counts(), get_dates(), and get_interval().

This package facilitates the manipulation of **incidence** objects by providing a set of handler functions for the most common tasks. The function subset() can be used for isolating case data from a specific time window and/or groups, while the [ operator can be used for a finer control to subset dates and groups using integer, logical or character vectors. This is accomplished by using the same syntax as for matrix and data.frame objects, i.e. x[i, j] where x is the **incidence** object, and i and j are subsets of dates and groups, respectively.

The function pool() can be used to merge several groups into one, and the function cumulate() will turn incidence data into cumulative incidence. To maximize interoperability, **incidence** objects can also be exported to either a matrix using get_counts() or a data.frame using as.data.frame(), including an option for a ‘long’ format which is readily compatible with *ggplot2* ( Wickham, 2016) for further customization of graphics.

In line with RECON’s development guidelines, the *incidence* package is thoroughly tested via automatic tests implemented using *testthat* ( Wickham, 2011), with an overall coverage nearing 100% at all times. We use the continuous integration services travis.ci and appveyor to ensure that new versions of the code maintain all existing functionalities and give expected results on known datasets, including matching reference graphics tested using the visual regression testing implemented in *vdiffr* ( Henry *et al*., 2018). Overall, these practices aim to maximise the reliability of the package, and its sustainable development and maintenance over time.

### Modeling utilities

Many different approaches can be used to model, and possibly derive predictions from incidence data (e.g. Cori *et al*., 2013; Nouvellet *et al*., 2018; Wallinga & Teunis, 2004), and are best implemented in separate packages (e.g. Cori *et al*., 2013). Here, we highlight three simple functionalities in *incidence* for estimating parameters via modeling or bootstrap and the two specialized data classes that are used to store the models and parameter estimates.

As a basic model, we implement the simple log-linear regression approach in the function fit(), which can be used to fit exponential increase or decrease of incidence over time by log-transforming case counts and applying a linear regression on these transformed data. The log-linear regression model is of the form *log*( *y*) = *r × t* + *b* where *y* is the incidence, *r* is the growth rate, *t* is the number of days since the start of the outbreak, and *b* is the intercept. This approach estimates a growth rate *r* (the slope of the regression), which can in turn be used for estimating the doubling or halving time of the epidemic, and with some knowledge of the serial interval, for approximating the reproduction number, *R*_0_ ( Wallinga & Lipsitch, 2007).

In the presence of both growing and decreasing phases of an epidemic, the date representing the peak of the epidemic can be estimated. In *incidence*, this can be done in two ways. The function estimate_peak() uses multinomial bootstrapping to estimate the peak, assuming that a) reporting is constant over time, b) the total number of cases is known, and c) the bootstrap never samples zero-incidence days. This function returns the estimated peak with a confidence interval along with the boostrap estimates. Alternatively, the function fit_optim_split() can be used to detect the optimal turning point of the epidemic and fit two separate models on either side of the peak. This is done by maximizing the combined mean adjusted *R* ^2^ value from the two models ( Figure 1, Figure 5).

The fit() function returns an **incidence_fit** object and the fit_optim_split() function returns an **incidence_fit_list** object, which is a specialized object designed to contain an unlimited number of (potentially nested) **incidence_fit** objects. While the *incidence* package returns **incidence_fit** objects containing log-linear models by default, they can be constructed from any model from which it’s possible to extract the growth rate ( *r*) and predict incidence along the model. Both object classes can be plotted separately or added to an existing epicurve using the function add_incidence_fit() ( Figure 5).

### Operation

The minimal system requirements for successful operation of this package is R version 3.1.

## Use cases

Two worked examples are used to demonstrate the functionality and flexibility of the *incidence* package. The first example illustrates how to compute and manipulate stratified weekly incidence directly from a line-list, while the second example shows how to import pre-computed daily incidence and fit a log-linear model to estimate growth rate ( *r*) and doubling time for the growing phase 1.

### Example 1: computing and manipulating stratified weekly incidence

In this first example, we use the dataset ebola_sim_clean in the *outbreaks* package, which provides a linelist for a fictitious outbreak of Ebola Virus Disease (EVD) that matches some key epidemiological properties (e.g. serial intervals, reproduction numbers) of the West African Ebola outbreak of 2014–2015 ( WHO Ebola Response Team *et al.*, 2014).

**1) Importing data**

First, we load the dataset ebola_sim_clean from the *outbreaks* package. The dataset contains 5,829 cases of 9 variables, among which the date of symptom onset ( $date_of_onset) and the name of the hospital ( $hospital) are used for computing the weekly epicurves stratified by hospitals.

library('outbreaks')

dat1 <- ebola_sim_clean$linelist
str(dat1, strict.width = "cut", width = 76)

## 'data.frame':      5829 obs.  of  9 variables:
##  $ case_id                   : chr  "d1fafd" "53371b" "f5c3d8" "6c286a" ...
##  $ generation                : int  0 1 1 2 2 0 3 3 2 3 ...
##  $ date_of_infection         : Date, format: NA "2014-04-09" ...
##  $ date_of_onset             : Date, format: "2014-04-07" "2014-04-15" ...
##  $ date_of_hospitalisation   : Date, format: "2014-04-17" "2014-04-20" ...
##  $ date_of_outcome           : Date, format: "2014-04-19" NA ...
##  $ outcome                   : Factor w/ 2 levels "Death","Recover": NA NA 2 ..
##  $ gender                    : Factor w/ 2 levels "f","m": 1 2 1 1 1 1 1 1 2 ..
##  $ hospital                  : Factor w/ 5 levels "Connaught Hospital",..: 2 ..

**2) Building the incidence object**

The weekly incidence stratified by hospitals is computed by running the function incidence() on the Date variable dat1$date_of_onset with the arguments interval = 7 and groups = dat1$hospital. The **incidence** object i.7.group is a list with class of **incidence** for which several generic methods are implemented, including print.incidence() and plot.incidence(). Typing **incidence** object i.7.group implicitly calls the specific function print.incidence() and prints out the summary of the data and its list components. The 5,829 cases (the total number of cases stored in the $n component) with dates of symptom onset ranging from 2014-04-07 to 2015-04-27 (spanning from 2014-W15 to 2015-W18 in terms of the ISO 8601 standard for representing weeks) are used for building the **incidence** object i.7.group. The $counts component contains the actual incidence for defined bins, which is a matrix with one column per group. Here $count is a matrix with 56 rows and 6 columns as groups by hospital with 6 factor levels are specified. The bin size in number of days is stored in the $interval component. In this example, 7 days suggests that weekly incidence is computed, while by default, daily incidence is computed with the argument interval = 1. The $dates component contains all the dates marking the left side of the bins, in the format of the input data (e.g. Date, integer, etc.). The $timespan component stores the length of time (in days) for which incidence is computed. The $cumulative component is a logical indication whether incidence is cumulative or not.

The generic plot() method for **incidence** objects calls the specific function plot.incidence(), which makes an incidence barplot using the *ggplot2* package. Hence, customization of *incidence* plot can benefit from the powerful graphical language from *ggplot2*.


library('incidence')
library('ggplot2')

# compute weekly stratified incidence
i.7.group <- incidence(dat1$date_of_onset, interval = 7, groups = dat1$hospital)
# print incidence object
i.7.group

## <incidence object>
## [5829 cases from days 2014-04-07 to 2015-04-27]
## [5829 cases from ISO weeks 2014-W15 to 2015-W18]
## [6 groups: Connaught Hospital, Military Hospital, other,
##  Princess Christian Maternity Hospital (PCMH), Rokupa Hospital, NA]
##
## $counts: matrix with 56 rows and 6 columns
## $n: 5829 cases in total
## $dates: 56 dates marking the left-side of bins
## $interval: 7 days
## $timespan: 386 days
## $cumulative: FALSE

# plot incidence object
my_theme <- theme_bw(base_size = 12) +
  theme(panel.grid.minor = element_blank()) +
  theme(axis.text.x = element_text(angle = 90, hjust = 1, vjust = 0.5, color = "black"))

plot(i.7.group, border = "white") +
  my_theme +
  theme(legend.position = c(0.8, 0.75))

**Figure 2.**
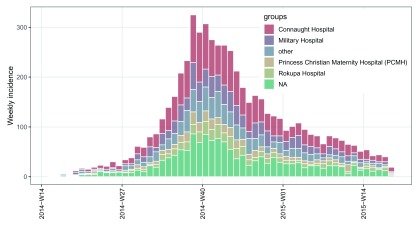
Weekly epicurves stratified by hospitals for the simulated outbreak of EVD.

Note that when weekly incidence is computed from dates, like in this example, the ISO 8601 standard weeks are used by default with the argument standard = TRUE in the incidence() function. Under this situation, an extra component of $isoweek is added to the **incidence** object i.7.group to store those weeks in the ISO 8601 standard week format “yyyy-Www”, and the $dates component stores the corresponding first days of those ISO weeks. Meanwhile the x-axis tick labels of the weekly *incidence* plot are in the ISO week format “yyyy-Www” (see Figure 2) rather than in the date format “yyyy-mm-dd” as the argument labels_iso_week in the plot() function is by default TRUE when plotting the ISO week-based **incidence** objects.

**3) Manipulate the incidence object**

In the above visualisation, it can be difficult to see what the dynamics were in the early stages of the epidemic. If we want to see the first 18 weeks of the outbreak in the four major hospitals, we can use the [ operator to subset the rows and columns, which represent weeks and hospitals, respectively, in this particular **incidence** object.

# plot the first 18 weeks, defined hospitals, and use different colors
i.7.sub <- i.7.group[1:18, grep("Hospital", group_names(1.7.group))]
hosp_colors <- c("#899DA4", "#C93312", "#FAEFD1", "#DC863B")
plot(i.7.sub, show_cases = TRUE, border = "black", color = hosp_colors) +
  my_theme +
  theme(legend.position = c(0.35, 0.8))

**Figure 3.**
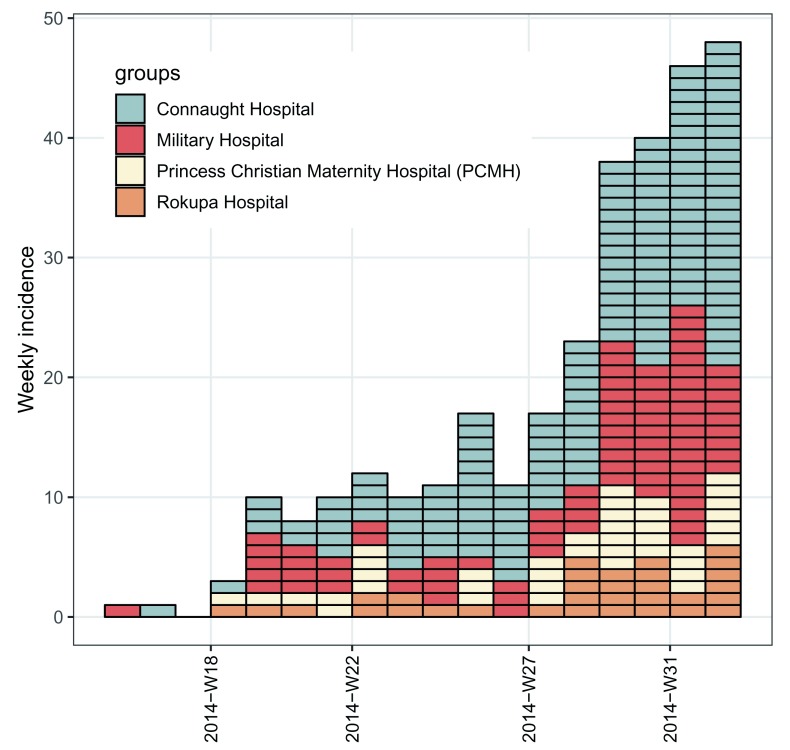
Weekly epicurves stratified by hospitals representing the first eight weeks of simulated outbreak of EVD.

Here, because of the few numbers of cases in the first few weeks, we have also highlighted each case using show_cases = TRUE ( Figure 3). We’ve also used a different color palette to differentiate between the subsetted data and the full data set.

As shown in Figure 2, the missing hospital name (NA) is treated as a separate group, resulting from the default of the argument na_as_group = TRUE in the incidence() function. This argument can be set to FALSE to not include data with missing groups in the object.

### Example 2: importing pre-computed daily incidence and fitting log-linear model

The datasets zika_girardot_2015 and zika_sanandres_2015 used in the second example are also from the *outbreaks* package. These datasets describe the daily incidence of Zika virus disease (ZVD) in, respectively, Girardot and San Andres Island, Colombia from September 2015 to January 2016. For details on these datasets, please refer to Rojas *et al*. (2016).

**Figure 4.**
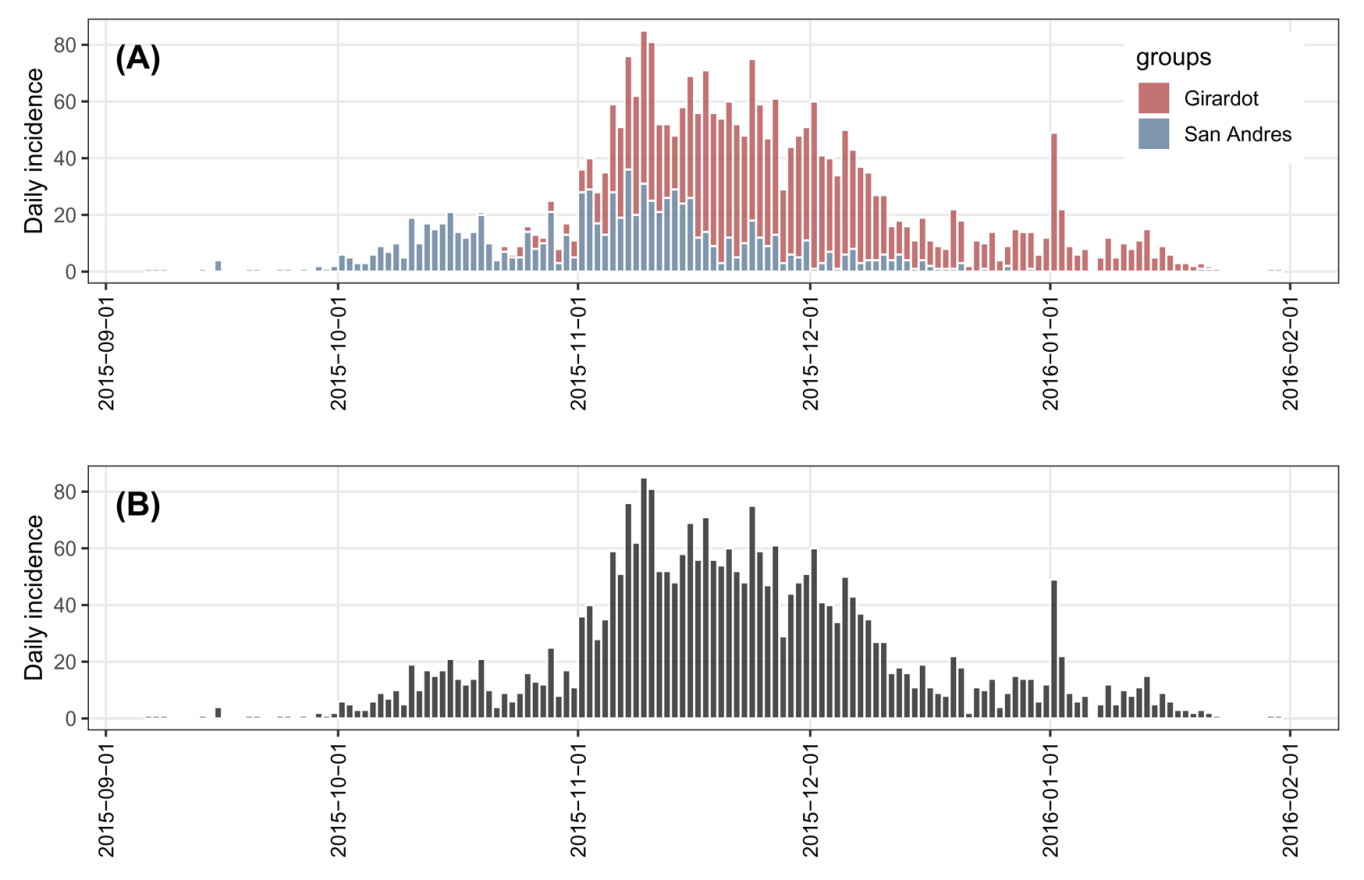
( **A**) stratified and ( **B**) pooled daily incidence plots of ZVD in Colombia, September 2015 to January 2016.

**1) Import pre-computed daily incidence**

zika_girardot_2015 and zika_sanandres_2015 are data frames with the same variables date and cases. In order to obtain a more complete picture of the epidemic dynamics of ZVD in Colombia, we merge these two data.frames into a single one, dat2, by variable date. As dat2 is already pre-computed daily incidence rather than a vector of dates such as those in example 1, we can directly convert it into an **incidence** object grouped by geographical locations, i.group, by using the as.incidence() function. This shows the flexibility of the *incidence* package in making **incidence** objects. Using the pool() function, the daily incidence stratified by locations, i.group, can be collapsed into an incidence object without groups, i.pooled. The stratified and pooled daily incidence plots of ZVD in Colombia are shown in Figure 4, from which we can see that the epidemic of ZVD occurred earlier in San Andres Island than in Girardot.

# preview datasets
head(zika_girardot_2015, 3)

##         date cases
## 1 2015-10-19     1
## 2 2015-10-22     2
## 3 2015-10-23     1

head(zika_sanandres_2015, 3)

##         date cases
## 1 2015-09-06     1
## 2 2015-09-07     1
## 3 2015-09-08     1

# combine two datasets into one
dat2 <- merge(zika_girardot_2015, zika_sanandres_2015, by = "date", all = TRUE)

# rename variables
names(dat2)[2:3] <- c("Girardot", "San Andres")

# replace NA with 0
dat2[is.na(dat2)] <- 0

# convert pre-computed incidence in data.frame into incidence object
# grouped by locations
i.group <- as.incidence(x = dat2[, 2:3], dates = dat2$date)

# pool incidence across two locations
i.pooled <- pool(i.group)
plot(i.group, border = "white")  + my_theme + theme(legend.position = c(0.9, 0.7))
plot(i.pooled, border = "white") + my_theme

As shown in Figure 4B, the pooled daily incidence in Colombia shows approximately exponential phases before and after the epidemic peak. Therefore, we fit two log-linear regression models around the peak to characterize the epidemic dynamics of ZVD in Colombia. Such models can be separately fitted to the two phases of the epicurve of i.pooled using the fit() function, which, however, requires us to know what date should be used to split the epicurve in two phases (see the argument split in the fit() function). Without any knowledge on the splitting date, we can turn to the fit_optim_split() function to look for the optimal splitting date (i.e. the one maximizing the average fit of both models) and then fit two log-linear regression models before and after the optimal splitting date.

library('magrittr')

fos <- fit_optim_split(i.pooled)
fos$split

## [1] "2015-11-15"

fos$fit

## <list of incidence_fit objects>
##
## attr(x, 'locations'): list of vectors with the locations of each incidence_fit object
##
## 'before'
## 'after'
##
## $model: regression of log-incidence over time
##
## $info: list containing the following items:
##   $r (daily growth rate):
##      before       after
##  0.06659200 -0.04813045
##
##   $r.conf (confidence interval):
##              2.5 %      97.5 %
## before  0.05869968  0.07448432
## after  -0.05440018 -0.04186071
##
##   $doubling (doubling time in days):
##   before
## 10.40887
##
##   $doubling.conf (confidence interval):
##           2.5 %   97.5 %
## before 9.305948 11.80836
##
##   $halving (halving time in days):
##    after
## 14.40143
##
##   $halving.conf (confidence interval):
##          2.5 %   97.5 %
## after 12.74163 16.55842
##
##   $pred: data.frame of incidence predictions (129 rows, 6 columns)

plot(i.pooled, border = "white") %>%
  add_incidence_fit(fos$fit) +
  my_theme

The returned object fos is a list with 4 components. The $split component suggests that the optimal splitting date is 2015-11-15. The $fit component is an **incidence_fit_list** containing two **incidence_fit** objects named ‘before’ and ‘after’. These each contain the information extracted from the fitted log-linear regression models. Printing the $fit component shows a daily growth rate *r* of 0.067 and its 95% confidence interval (CI) ([0.059, 0.074]), and a doubling time of 10.4 days (95% CI, [9.31, 11.8]) during the first phase, and a daily decreasing rate *r* of -0.048 (95% CI, [-0.054, -0.042]), and a halving time of 14.4 days (95% CI, [12.7, 16.6]) during the second.

**Figure 5.**
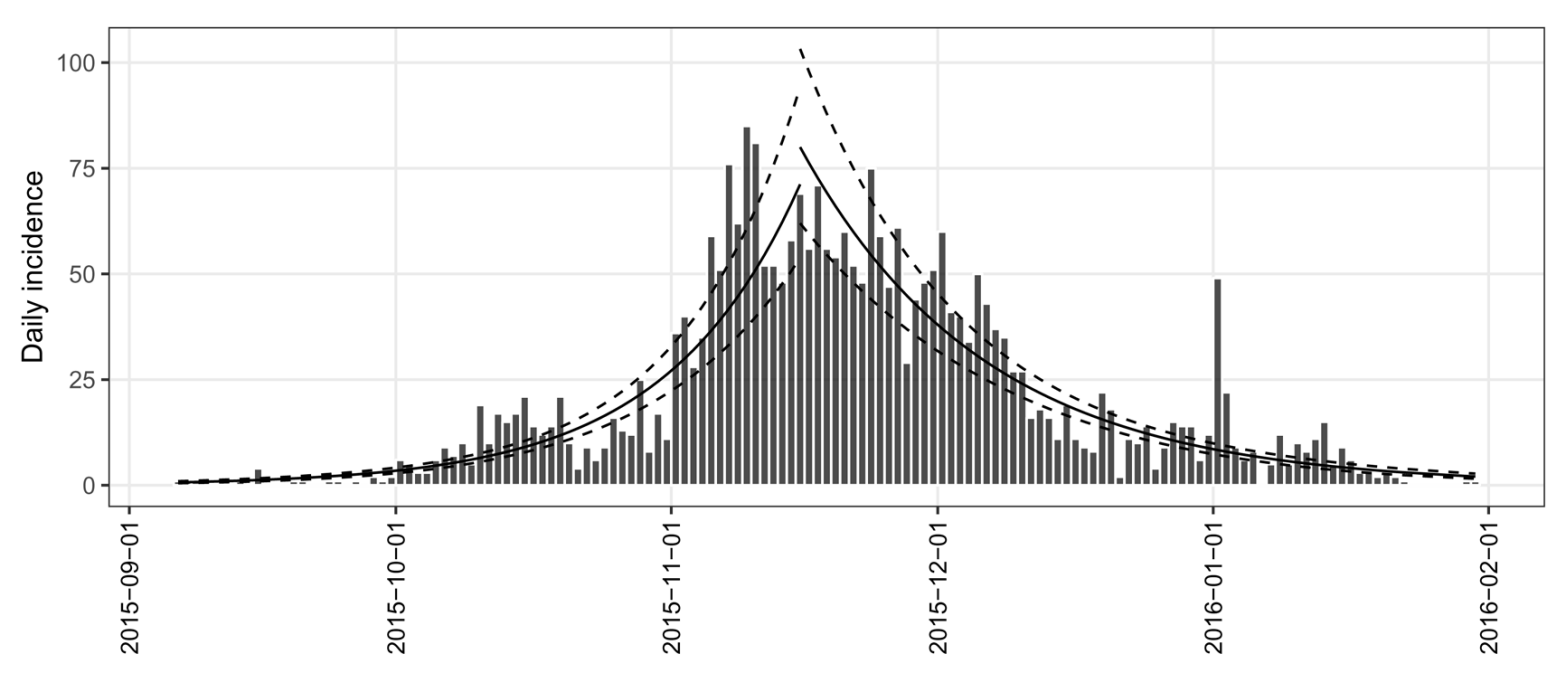
Fit two log-linear regression models, before and after the optimal splitting date.

The predictions and their 95% CIs from the two **incidence_fit** objects, ‘before’ and ‘after’, can be added to the existing incidence plot of i.pooled using the piping-friendly function add_incidence_fit(). As shown in Figure 5, based on visual comparison of models and data, these two log-linear regression models provide a decent approximation for the actual dynamics of the epidemic (adjusted *R* ^2^ = 0.83 and 0.77 for the increasing and decreasing phases, respectively).

## Conclusion

This article has described the package *incidence* and its features—which include three lightweight data classes and utilities for data manipulation, plotting, and modeling. We have shown that an **incidence** object can flexibly be defined at different datetime intervals with any number of stratifications and be subset by groups or dates. The most important aspects of this package are use-ability and interoperability. For both field epidemiologists and academic modellers, the data received are often in the form of line-lists where each row represents a single case. We have shown that these data can easily be converted to an **incidence** object and then plotted with sensible defaults in two lines of code.

We have additionally shown that because the data are aggregated into a matrix of counts, it becomes simple to perform operations related to peak-finding, model-fitting, and exportation (e.g. using as.data.frame()) into different formats. Thus, because it has built-in tools for aggregation, visualisation, and model fitting, the *incidence* package is ideal for rapid generation of reports and estimates in outbreak response situations where time is a critical factor.

## Software availability

*incidence* available from: https://www.repidemicsconsortium.org/incidence Code to reproduce all figures can be found by running demo ("incidence-demo", package = "incidence") from the R console with the incidence package installed.

Source code available from: https://github.com/reconhub/incidence

Archived source code as at time of publication: https://doi.org/10.5281/zenodo.2540217 ( Jombart *et al*., 2019)

Software license: MIT

## Data availability

### Underlying data

Datasets used in the worked examples are from the *outbreaks* package:

ebola_sim_clean: https://github.com/reconhub/outbreaks/blob/master/data/ebola_ sim_clean.RData

zika_girardot_2015: https://github.com/reconhub/outbreaks/blob/master/data/zika_ girardot_2015.RData

zika_sanandres_2015: https://github.com/reconhub/outbreaks/blob/master/data/zika_sanandres_2015.RData

## Footnotes

1 Negative values of *r* in incidence are reported as halving times instead of doubling times and decreasing phase instead of growing phase
