# Supplementary material for: Epidemic curves made easy using the R package incidence
Source: F1000Res. 2019 Jan 31;8:139. [Version 1] doi: 10.12688/f1000research.18002.1 (PMC6509961; doi:10.12688/f1000research.18002.1)
Supplement: Peer review history

# Peer review: Reviewer response for version 1

**DOI:** 10.5256/f1000research.19688.r48153

**Published:** 2019-05-07

**Authors:** Bertrand Sudre

**Affiliations:** 1 European Centre for Disease Prevention and Control (ECDC), Solna, Sweden

**Major comments**

 The proposed package is addressing some of the topmost descriptive elements of any epidemiological data set, namely a systematic time-place-person description. With regards to epidemiological curves, a limited number of dedicated packages addressing these aspects were available at the time of this package release (mainly: epitools, https://cran.r-project.org/web/packages/epitools/epitools.pdf  [last update: October 26, 2017] and EpiCurve, https://cran.rstudio.com/web/packages/EpiCurve/EpiCurve.pdf [last update: April 24, 2018]). The alternative was a tailor-made, and time consuming, customization based on existing dedicated packages (for instance using customized bar charts geoms from ggplot2). While the data storage and representation is well addressed by the authors, the proposed package offers: i) some basic utilities for outbreak description across time, ii) basic tool for outbreak modelling and iii) a standard for data storage to enhance interoperability between released projects and packages from the R epidemic consortium. In the introduction a short overview of the alternative tools mentioned above should be provided to the reader, together with the new added-values of the current package. It would be an asset in order to ensure a benchmarking analysis with pre-existing resources.

 “Here, we introduce *incidence*, an R package developed as part of the toolbox for epidemics analysis of the R Epidemics Consortium (RECON) which aims to fill this gap. In this paper, we outline the package’s design and illustrate its functionalities using a reproducible worked example.”

 According to RECON website: package incidence corresponds to “Computation, handling, visualisation and simple modelling of incidence”. It is honourable that the name of a package “incidence” is the choice of the creators of course. It is true that incident cases are all individuals who change to non-disease status from disease, so in this way “incidence” could refer to the occurrence of new cases. In recent modelling papers, the term incidence has been associated to count time series under the same approach, so-called “incidence time series” ^1^.

 Nevertheless, the current name can be misleading for a certain number of epidemiologists. The reason is that in epidemiology the term incidence is traditionally associated to a measure of morbidity, so-called 'Incidence proportion' (or attack rate or risk; for more information see: https://www.cdc.gov/ophss/csels/dsepd/ss1978/lesson3/section2.html). The latter comprised the numerator (= count of case used for a raw epi-curve) but as well a denominator representing the population at risk during the selected time interval. At the first glance, the target audience reading the package name might believe that the package is dedicated to incidence calculation rather than epidemiological curve graphic representation and some basic modelling utilities. Indeed, the package is presented as being able to compute, handle and visualize time-related count data through epi-curve and additional derived features which are not related to measure of incidence (proportion) strictly speaking.

 You may wish to consider adding such features and include new capabilities to this package which can cover calculation/representation of incidence in epidemiology. For instance, adding a slot for population data to populate the denominator of an incidence proportion calculation. This can be completed by dedicated graphic outputs with points for each incidence values and line through all data points (geom line). Factor-specific incidence rate (and corresponding CI) can be considered as further extensions (factor: sex-, age-, or any other factor). The advantage is the possibility to overlay and compare several incidence line charts coming for different locations (e.g. attack rate for different health districts or for several population group). It is a suggestion and it is acceptable that the authors would keep the package focus on basic utilities and epicurve representation.

 With regards to the structure of the article, it would be easier to start with an example based on simple line listing (see comment on figure 1) with simple epicurve without stratification (with different timing hour, day. week), then move to more complex representation (stratification with various colour in the legend; +/- facetting), and then an example of tailor-made polished figure (see code below). In doing so the figure 1 which would start for a line-list format would be easier to understand.

**Minor comments**

**Section:** **Author’s affiliation**

 Comment 1:

- “Department of Infectious Disease Epidemiolog y , School of Public Health, Imperial" - Space to remove after epidemiology
- “Epidemiological Modelling and Analysis (SACEMA ),, Stellenbosch University, Stellenbosch” - Double coma to remove

**Section: Introduction**

 Comment 2:

- “Responses to infectious disease epidemics use a growing body of data sources to inform decision making (Cori *et al*., 2017; Fraser *et al*., 2009; WHO Ebola Response Team *et al*., 2014; WHO Ebola Response Team *et al*., 2015). While new data—such as whole genome pathogen sequences—are increasingly useful complements to epidemiological data (Gire *et al*., 2014), epidemic curves—which describe the number of new cases through time (incidence)—remain the most important source of information, particularly early in an outbreak.” - Rephrase avoiding four "—" in the same sentence.

Partial genome sequencing is useful to complement epidata/infect. disease epi. If you wish to highlight the WGS/extensive sequencing, consider several aspects:

- Confirm change in infectiousness or epidemiology (CFR, change in clinical presentation, shift in risk group.)
- Relationships between cases (mapping Lassa, Ebola transmission chains ...)
- Emergence and patterns of transmission (vector, host, reservoir)

Suggested examples from the recent literature:

- Kraemer MUG, Cummings DAT, Funk S, Reiner RC, Faria NR, Pybus OG, Cauchemez S. Reconstruction and prediction of viral disease epidemics. Epidemiol Infect. 2018  Nov 5:1-7. doi: 10.1017/S0950268818002881. [Epub ahead of print] PubMed PMID: 30394230; PubMed Central PMCID: PMC6398585. ^2^               
- Siddle KJ *et al*. Genomic Analysis of Lassa Virus during an Increase in Cases in Nigeria in 2018. N Engl J Med. 2018 Nov. ^3^ 
- Kafetzopoulou LE *et al*, Metagenomic sequencing at the epicenter of the Nigeria 2018 Lassa fever outbreak. Science. 2019 Jan 4;363(6422):74-77. ^4^

Comment 3:

- “which describe the number of new cases through time (incidence)—remain” -Proposed change of 'through' to 'over'

Comment 4:

- “important source of information, particularly early in an outbreak.” - Not only. This can be helpful to look at the magnitude and pattern (recurrent environmental sources, detect outliers ...).

Comment 5:

- “Specifically epidemic curves(often” - Missing space.

Comment 6:

- “[…] often referred to as ‘epicurves’) represent the number of new cases […]” - New cases = incident case in epidemiology. Might be more accurate to say, "incident cases of a disease” and add within bracket “(corresponding to the vertical y-axis)”.
- “[…] per time unit based […]” - Consider 'time interval' instead of 'unit' as ‘unit’ can be misunderstood (e.g. unit = hour, min, sec). The interval is at the user's discretion (3 hours = 'time interval’).
- “[…]: on the date or time of symptom onset” - Consider replacing symptoms by “time of illness onset among cases” (and add within bracket “(corresponding to the vertical x-axis)”.

Comment 7:

- “provide a simple, visual outline of epi­demic dynamics, which can be used for assessing the growth or decline of an” - That is the main purpose in practice. You may consider being more assertive, by replacing "can" with "is". Very well-known added-values. No need for six references to support this statement.

Comment 8:

- “[…] by a range of modelling techniques for short-term forecasting (Cori *et al*., 2013; Funk *et al*., 2018; Nouvellet *et al*., 2018; Viboud *et al*., 2018)” - Could you please consider developing with few sentences pointing to ad hoc examples in relation with the features of the cited references.

Comment 9:

- “[…] as well as in outbreak detection algorithms from syndromic surveillance data” - Consider more references that are recent. Note such analytical frameworks are not restricted to syndromic surveillance but to any regularly collected count data from epidemiological time-series in health surveillance system (syndromic or not).

Comment 10:

- “[…] But despite the existence of packages dedicated to time series analysis (Shumway & Stoffer, 2010) as well as surveillance data (Höhle, 2007)” - Time series analysis is a generic term, consider “time series of epidemiological data” to be more accurate. Numerous time-series packages are available for count data that can be "re-used" for human epi.

Comment 11:

- “a lightweight and *well-tested *package solely dedicated to building, handling and plotting epidemic curves directly from linelist data (e.g. a spreadsheet where each row represents an individual case) is still lacking.” - Perhaps “lightweight” is something relative in package development and might be changed. More importantly, as mentioned above, please, consider mentioning other previous packages supporting specifically epi-curve creation (for instance: epitools, EpiCurve). These packages can manage aggregated/no aggregated data with and without factor. Current presented package has an added-value is its interoperability, the presence of simple modelling tools and further graph customization. Stricto sensu, epicurve were able to be done in R form user customization of ggplot2 and cited package.

**Section: Methods**

 Comment 12:

- “[...] some additional facilities for basic models of incidence over time.” - Consider “additional features” instead.

Comment 13:

- “[...] an indication of whether incidence is cumulative or not” - Consider “whether the case count is cumulative”.

Comment 14:

- “[…] representing dates including Date and POSIXct objects”. - The EpiCurve package supports hourly data. This intends to cope with some peculiar situation in which an hourly epi-curve can be of interest (acute food intoxication or environmental source). Could give an example with hourly data (it should be fairly easy as POSIXct is date format in the ggplot2 framework). See an example here: https://www.cambridge.org/core/journals/epidemiology-and-infection/article/unusually-high-illness-severity-and-short-incubation-periods-in-two-foodborne-outbreaks-of-salmonella-heidelberg-infections-with-potential-coincident-staphylococcus-aureus-intoxication/FED73E4BCEFC109DA327F2909B80BBA7/core-reader

Comment 15:

- “The dates are aggre­gated into counts based on the user-defined interval representing the number of days for each bin.”- Consider 'user-defined time interval' instead of user-defined interval.
- “number of days for each bin” - “days” is too restrictive. It is just the chosen time interval, it can be the number of weeks, etc.

Comment 16:

- “also accepts a groups argument which can be used to obtain stratified incidence“ -Consider changing stratified incidence by “epidemiological curve".

Comment 17:

- “The basic elements of the incidence object can be obtained by the accessors get_counts(), get_dates(), and get_interval()." - Please, number the number of basic elements for clarity purpose.

Comment 18:

- “The function subset() can be used for isolating case data from a specific time window and/or groups, while the [ operator can be used for a finer control to subset dates and groups using integer, logical or character vectors.” - If several functions are to be presented, it is easier to use bullet points to structure the reading. 
- Consider the removal 'for isolating case data from a specific' and changing with 'to define'.
- “[“ change to 'indexing operator, to follow the classical denomination https://cran.r-project.org/doc/manuals/r-release/R-lang.pdf.

Comment 19:

- “Figure 1. Generalized workflow from incidence object construction to modeling and visualization.” - The first example in the paper is using a line-list format as data inputs but showed a stratified graphic. To be consistent and easier to follow for the reader, this figure illustrates the flow of such of data type, knowing that line-list is the primary source of epidemiological surveillance. A solution present in the figure is the two types of data inputs (non-aggregated/aggregated count). It would be easier, from a reader point of view, to capture the data flow from the line-lit to the final products proposed by this package.

Comment 20:

- “The function pool() can be used to merge several groups into one,” - Consider ending the sentence and explaining what it does.
- “and the function cumulate() will turn incidence data into cumulative incidence” - Consider changing “cumulative incidence” to “cumulative count of cases”.

Comment 21:

- […]: an option for a ‘long’ format which is readily compatible with *ggplot2 *(Wickham, 2016) for further customization of graphics.” - Would it be possible to mention how date format is exported? This might good to elaborate a bit more about date and user's customization with ggplot2. This can be addressed later in the manuscript (see proposal for a theme).

Comment 22:

- “In line with RECON’s development guidelines, the *incidence *package is thoroughly” - Add a hyperlink/ref. to the website: https://www.repidemicsconsortium.org/resources/guidelines/

**Section: Modelling utilities**

 Comment 23:

- “Here, we highlight three simple functionalities in *incidence *for estimating parameters via modelling or bootstrap and the two specialized data classes that are used to store the models and parameter estimates.” - Consider structuring the following section according to the five elements mentioned (=three functions [ fit() , estimate_peak() , fit_optim_split()] and two specialized data classes) using for instance bullet points/subtitles. For each function, the goal, data input, statistical methods and output object(s) can be grouped in single section.

Comment 24:

- “we implement the simple log-linear regression approach in the function fit(), which can […]" - Please add more information about the structure of the 'incidence_fit objects containing log-linear models'

Comment 25:

- “[…] fit exponential increase or decrease of incidence over time by log-transforming case counts … “ - Can be simplified to “fit exponential increase or decrease using a linear regression over time on log-transformed case counts…“.

Comment 26:

- “where *y *is the incidence, *r *is the growth rate, *t"* - Replace “incidence” with number of new case/incident case.

Comment 27:

- “serial interval” - Consider adding “serial interval of the infectious agent”

Comment 28:

- “uses multinomial bootstrapping to estimate the peak, assuming” - Some explanation about the method and references would be desirable.

Comment 29:

- “Both object classes can be plotted separately or added to an existing epicurve using the function add_incidence_fit() (Figure 5)." - The customization of the epicurve is well described. However, it is not mentioned how to change the layout of the model outcome and confidence interval. Indeed, some users might wish to use an alternative ggplot2 geometric object such as geom_range with a shaded grey semi-transparent band instead of two dotted lines. It would an added-value to provide some capacities or explanation and an example of customization of the layout of the “incidence_fit objects”.

**Section: Use cases**

 Comment 30:

- “Two worked examples are used to demonstrate the functionality and flexibility of the *incidence *package. The first example illustrates how to compute and manipulate stratified weekly incidence directly from a line-list”. - Consider “The first example illustrates how to create directly from a line-list incidence object in order draw an epicurve of the weekly number of cases with or without stratification on patient characteristics”.

Comment 31:

- “while the second example shows how to import pre-computed daily incidence and fit a log-linear model to estimate growth rate ( *r*) and doubling time for the growing phase.” - Footnote to be included in the section about the function for more clarity.

**Example 1: computing and manipulating stratified weekly incidence**

 Comment 32:

- “The weekly incidence stratified by hospitals is computed by running the function incidence() on the Date vari­able dat1$date_of_onset with the arguments interval = 7 and groups = dat1$hospital." - Consider rephrasing. For instance: “the epicurve with the weekly number of cases by hospital can be computed from the line listing dataframe object (dat1) using the function incidence() on i) the date vari­able (dat1$date_of_onset), ii) by specifying the argument interval of seven days in order to aggregate the number case per week (interval = 7) and iii) including a the line-listing variable for stratification in the argument groups, in this case the hospital name (groups = dat1$hospital).

Comment 33:

- “Here $count is a matrix with 56 rows and 6 columns as groups by hospital” - Missing s at the end of $counts.

Comment 34:

- “The generic plot() method for incidence objects calls the specific function plot.incidence(), which makes an incidence barplot using the *ggplot2 *package. Hence, customization of *incidence *plot can benefit from the powerful graphical language from *ggplot2*.” - A short explanation and command line to explain how to access the code of the method would be welcome (notably the plot). This would help users to understand which ggplot2 geometric object(s) is used for the bar plot and incidence_fit lines. This would be an asset to understand how to proceed with further customization (within the aesthetic, theme or faceting specifications). This can be proposed at the end through several examples.

Comment 35:

- # plot incidence object
  my_theme <-theme_bw(base_size = 12) +
  theme(panel.grid.minor = element_blank()) +
  theme(axis.text.x = element_text(angle = 90, hjust = 1, vjust = 0.5, color = "black")) plot(i.7.group, border = "white") + my_theme + theme(legend.position = c(0.8, 0.75))

The current example assumes that all users are familiar with ggplot2, notably how to customize the non-data components through the theme. We might suggest to introduce what is a theme in ggplot2 and what it does, for instance “Themes allows modification (content and layouts) of non-data components such as titles, axis labels, legends (position and aspect …), graphics grid lines and backgrounds (Modify components of a theme; ref: https://ggplot2.tidyverse.org/reference/theme.html)”.

 In addition, mention that theme specifications can “overwrite” some layout specification in the other part of the ggplot2 function. The use in this example of theme_bw appears to clarify what the default built-in theme is (complete themes: https://ggplot2.tidyverse.org/reference/ggtheme.html , other themes https://cran.r-project.org/web/packages/ggthemes/ggthemes.pdf).

 To help the reader, you might consider the arguments as displayed in “Modify components of a theme” order as much as possible:

- my_theme <-theme_bw(base_size = 12) +
  theme (
  panel.grid.minor = element_blank,
  axis.text.x = element_text(angle = 90, hjust = 1, vjust = 0.5, color = "black"),
  legend.position = c(0.8, 0.75)
  )

For this first example, consider setting the legend outside of the main graphic frame, especially for the first figure (instance legend.position="bottom", legend.box = "horizontal").

 For the axis labels, the Y is family the “Num. of case of EVD. per week” in the Figure 2.

 Comment 36:

- i.7.sub <- i.7.group[1:18, grep("Hospital", group_names(1.7.group))]
  hosp_colors <-c("#899DA4", "#C93312", "#FAEFD1", "#DC863B")
  - Add an intermediate line to illustrate that in this period selection (e.g. print i.7), cases are coming from four hospitals, to hosp_colors would a four colours vector. Keep the legend out of the main graphic frame (top or bottom).

Comment 37:

- “Here, because of the few numbers of cases in the first few weeks, we have also highlighted each case using show_ cases = TRUE (Figure 3)”. - The figure is displaying the individual case which is the standard representation of an epicurve according to number of training programs (US CDC, EPIET …). This is of primary importance in outbreak investigation to able to delineate case number in an easy-to-read visual and often stratified representation by case characteristics (sex, exposure, location …).  This type of representation is adopted by default in package EpiCurve (see https://cran.r-project.org/web/packages/EpiCurve/vignettes/EpiCurve.pdf) as well as US CDC training example of epicurve ( https://www.cdc.gov/training/quicklearns/createepi/). Representation of case as square can be achieved with the current package as illustrated in at the end of the package vignette (see: vignette ("customize_plot", package="incidence") in the section Applying the style of European Programme for Intervention Epidemiology Training (EPIET). It’s understandable that square form can be changed in case of a large dataset making the size of the intervals for the x- and y- axes different.
  It would be an asset for the target audience and the current manuscript to contain a full example of an epicure with case made under square representation (somehow close to the example cited above in the vignette) combined with a full theme following simple and standard representation.

Comment 38

- Consider change in the layout of Figure 2: 'Number of EVD cases stratified by hospital ... between week XX and week YY'.
  Y axis title: 'Number of cases'
  X axis title: 'Week of onset of illness'
  X axis ticks should be made visible for all weeks even the label displayed for any other week.

Comment 39:

- Would it be possible to give some more information to which ggplot2 geometry are used when show_ cases = TRUE is specified? This is of importance to provide the reader with a clue about how both borderline and colour content of each square can be further customized.

Comment 40:

- As standard the label of the X axis time interval is displayed on the left side of the bin. Formally, the ideal position would be right below the bin (as illustrated in the CDC example above and numerous of published epicurve). In the EpiCurve package, the standard representation is not ideal as the X axis tick is right in the middle of the bin. The position of the label can be customized by the user, but would it be possible to look at the option to place the time label under each bin instead of to the left bin tick mark (see an example of ad hoc customization below). Of note, such label position should support figure export resizing.

Comment 41:

 Figure 4. (A) stratified and (B) pooled daily incidence plots of ZVD in Colombia, September 2015 to January 2016.

 Please consider comments made above for the other figure on figure title and axis labels:

- “Epicurve of the daily number of Zika virus disease between Sep and Jan 2016”. Panel (A) stratified by location and (B) pooled across locations. “
- Y axis title: 'Number of cases'
- X axis title: ' Week of onset of illness'

Some readers might wonder if the faceting capabilities of ggplot2 would be supported or not. An evident alternative is to prepare separated epicurve for the both locations and further combined them using specific package (ggarrange for instance). Would it be possible to add information on this point, and if supported, provide an alternative presentation of the both epicurves using two vertically aligned panels?

 Comment 42:

- “Without any knowledge on the splitting date, we can turn to the fit_optim_split() function to look for the optimal splitting date”. - Could you please reconsider the phrasing as “Without any knowledge on the splitting date” which seems not logical when looking retrospectively to an outbreak epicurve and visually identifying the peak of the epidemic wave.

Comment 43:

- library('magrittr') - Provide a short explanation about the dependency(ies) with this package.

Comment 44:

- “The predictions and their 95% CIs from the two incidence_ft objects, ‘before’ and ‘after’, can be added to the existing incidence plot of i.pooled using the piping-friendly function add_incidence_fit().” - Provide more explanation on “the piping-friendly function” aspect. Provide an example of layout customization of the two log-linear regression models (linetype, colour, size).

Comment 45:

- Please find below a proposal for an advanced and custom-made layout epicurve representation using the Zika dataset. In this example, some of the important layout feature of an epicurve are available: i) no space around the limits of both x and Y axes, ii) representation of each case with a square, iii) x label under each bin and make the graphic as light as possible based on Tufte’s advice (less grid as possible, visible labels …).
- In the graphic below, an incidence_fit object can be added for the pooled location with a specific customization to give a complete overview to the package capabilities.

 

**Working example based on Zika diease dataset:**

****

 head(zika_girardot_2015, 20)

 head(zika_sanandres_2015, 20)

 dat2 <- merge(zika_girardot_2015, zika_sanandres_2015, by = "date", all = TRUE) # # combine two datasets into one

 names(dat2)[2:3] <- c("Girardot", "San Andres") # rename variables

 dat2[is.na(dat2)] <- 0 # replace NA with 0

 i.group <- as.incidence(x = dat2[, 2:3], dates = dat2$date) # convert pre-computed incidence in data.frame into

 str(i.group)

 # Graphic

 i.group_zoom_in <- i.group[10:60]

 plot(i.group_zoom_in, n_breaks = nrow(i.group_zoom_in),  border = "grey90", show_cases=TRUE) +

   theme_bw() +

   scale_y_continuous(expand = c(0, 0), limits=c(0,max(i.group_zoom_in$counts +1))) +

   scale_x_date(date_breaks = "1 day", date_labels = "%b %d",expand = c(0, 0), limits= c(as.Date(min(i.group_zoom_in$dates)) , as.Date(max(i.group_zoom_in$dates+1)))) +

  #  scale_fill_discrete(name = "Location:") +

   #scale_x_continuous(expand = c(0, 0),limits= c(as.Date(min(zomm_in$dates)) , as.Date(max(zomm_in$dates)))) +

   labs(title = "Number of Zika disease cases",

        subtitle = "Girardot and San Andres municipalities, Colombia. Period: 6 Sep to 11 Oct 2015." ,

        x="Week of onset of illness",

        y="Number of cases",

        fill="Municipalities:") +

   theme(panel.border = element_rect(colour = "white"),

         panel.grid.major.y = element_line(colour = "grey70", linetype="dotted", size =0.5),

         axis.line = element_line(colour = "black", size = 0.7),

         axis.text.x = element_text(angle = 45, hjust = 0.8, size = 6),

         axis.ticks.x =  element_line(size = rel(2)),

         axis.ticks.y =  element_line(size = rel(2)),

         axis.title.y = element_text(margin=margin(0,10,0,0), size=10),

         axis.title.x = element_text(margin=margin(10,0,0,0), size=10),

         plot.title =  element_text(size = 12,face = "bold"),

         plot.subtitle =  element_text(size = 9),

         legend.position="bottom",

         legend.box = "horizontal") +

   coord_equal()

 Link to plot available here. 

**Is the rationale for developing the new software tool clearly explained?**

 Yes. The rational is sounds and the new package “Incidence” is fulfilling the objectives cited. It allows streamlining the manipulation and representation for epidemiological data for epidemiological representation. It expected that this new package would reach a wide audience of epidemiologists and epidemiological data analysts working with R.

**Is the description of the software tool technically sound?**

 Yes. However, some clarifications proposed in the detailed review would enhance the description of the software tool features.

**Are sufficient details of the code, methods and analysis (if applicable) provided to allow replication of the software development and its use by others?**

 Overall, yes. Minor adjustments are proposed to allow reader to better access code (e.g. provide example to access full code of incidence (S3) class incidence  and its subsequent methods (plot, …)) and offer additional  clarification to improve graphic customization (notably around the incidence_fit object and further integration of ggplot2  faceting functionality).

**Is sufficient information provided to allow interpretation of the expected output datasets and any results generated using the tool?**

 Yes. Several practical and realistic examples are provided by the authors. All examples were tested and further tests with epidemiological additional datasets were conducted and allow to reproduce accurately tool behaviours. In addition to the paper review, the R package documentation (description file, recent reference manual and vignettes) were thoroughly reviewed to assess any discrepancies between the peer-review publication and package documentation on CRAN.

**Are the conclusions about the tool and its performance adequately supported by the findings presented in the article?**

 Yes.

I have read this submission. I believe that I have an appropriate level of expertise to confirm that it is of an acceptable scientific standard, however I have significant reservations, as outlined above.

## References

[1] CoriAFergusonNMFraserCCauchemezS: A new framework and software to estimate time-varying reproduction numbers during epidemics. *Am J Epidemiol*.2013;178(9) : 10.1093/aje/kwt133 1505-12 10.1093/aje/kwt133 24043437PMC3816335

[2] KraemerMUGCummingsDATFunkSReinerRCFariaNRPybusOGCauchemezS: Reconstruction and prediction of viral disease epidemics. *Epidemiol Infect*.2018; 10.1017/S0950268818002881 1-7 10.1017/S0950268818002881 30394230PMC6398585

[3] SiddleKEromonPBarnesKMehtaSOguzieJOdiaISchaffnerSWinnickiSShahRQuJWohlSBrehioPIruolagbeCAiyepadaJUyigueEAkhilomenPOkonofuaGYeSKayodeTAjogbasileFUwanibeJGayeAMomohMChakBKotliarDCarterAGladden-YoungAFreijeCOmoregieOOsiemiBMuoebonamEAirendeMEnigbeREboBNosamiefanIOluniyiPNekouiMOgbaini-EmovonEGarryRAndersenKParkDYozwiakNAkpedeGIhekweazuCTomoriOOkogbeninSFolarinOOkokherePMacInnisBSabetiPHappiC: Genomic Analysis of Lassa Virus during an Increase in Cases in Nigeria in 2018. *New England Journal of Medicine*.2018;379(18) : 10.1056/NEJMoa1804498 1745-1753 10.1056/NEJMoa1804498 PMC618118330332564

[4] KafetzopoulouLPullanSLemeyPSuchardMEhichioyaDPahlmannMThielebeinAHinzmannJOestereichLWozniakDEfthymiadisKSchachtenDKoenigFMatjeschkJLorenzenSLumleySIghodaloYAdomehDOlokorTOmomohEOmiunuRAgbukorJEboBAiyepadaJEbhodaghePOsiemiBEhikhametalorSAkhilomenPAirendeMEsumehRMuoebonamEGiwaREkanemAIgenegbaleGOdigieGOkonofuaGEnigbeROyakhilomeJYerumohEOdiaIAireCOkonofuaMAtafoRTobinEAsogunDAkpedeNOkokherePRafiuMIraoyahKIruolagbeCAkhidenoPEramehCAkpedeGIsiborENaidooDHewsonRHiscoxJVipondRCarrollMIhekweazuCFormentyPOkogbeninSOgbaini-EmovonEGüntherSDuraffourS: Metagenomic sequencing at the epicenter of the Nigeria 2018 Lassa fever outbreak. *Science*.2019;363(6422) : 10.1126/science.aau9343 74-77 10.1126/science.aau9343 PMC685537930606844

# Peer review: Reviewer response for version 1

**DOI:** 10.5256/f1000research.19688.r45525

**Published:** 2019-04-25

**Authors:** Quirine ten Bosch

**Affiliations:** 1 Quantitative Veterinary Epidemiology, Department of Animal Sciences, Wageningen University and Research Centre, Wageningen, The Netherlands

The authors introduce a package that aids in processing linelist data ( *i.e., *in which each row represents a case) for building, handling, and plotting epidemic curves. This tool fills a gap within the larger epidemics toolbox of the R Epidemics Consortium in ‘getting the basics right’, or otherwise put, getting data common to outbreak situations in the right format for further analysis.

 I agree with the authors that this is a helpful tool. This is particularly true during outbreaks, when linelist data need to be processed quickly and disseminated to relevant actors. The procedures are straightforward and well presented. The examples given in this work are well chosen and provide a good starting point for working with this package. I only have a few comments pertaining to i) the loglinear model implementation and presentation and ii) the integration with other packages.

1. While the package is meant to focus on processing and visualization of data, the authors have added a modeling-capability that estimates epidemic growth rates. The respective function contains an option for estimating the peak of the outbreak and fit the exponential increase or decrease during the epidemic. Basing the time window for exponential growth on the timing of the peak will underestimate the growth rate as growth will no longer be exponential just before the peak of the epidemic. A more careful description in the MS is needed to highlight this and other shortcomings of this approach. Other methods to estimate the best time window, such as those used in the R0-package ^1^, could prove helpful and be implemented in the package. Further, the choice of fitting the growth rates to the aggregated data rather than the two distinct regions (Girardot and San Andres) is a bit uncomfortable. Particularly so because, as the authors acknowledge, there seem to be two quite distinct outbreaks.
2. The authors highlight the gap within the epidemics toolbox is filled. It would be nice to see a little bit more context on what packages can easily work with their data structures and what analyses can hence readily be done.

Minor comments:

- Page 7: group_names(1.7.group) should be i.7.group

I have read this submission. I believe that I have an appropriate level of expertise to confirm that it is of an acceptable scientific standard.

## References

[1] ObadiaTHaneefRBoëllePY: The R0 package: a toolbox to estimate reproduction numbers for epidemic outbreaks. *BMC Med Inform Decis Mak*.2012;12: 10.1186/1472-6947-12-147 147 10.1186/1472-6947-12-147 23249562PMC3582628

# Peer review: Reviewer response for version 1

**DOI:** 10.5256/f1000research.19688.r45526

**Published:** 2019-04-03

**Authors:** Benjamin M. Bolker

**Affiliations:** 1 Departments of Mathematics and Statistics and Biology, McMaster University, Hamilton, ON, Canada

The authors have written an R package that does some useful tasks in aggregating and manipulating incidences and plotting them. For the most part the paper is clearly written and technically correct, and the package seems well-constructed. The abstract says that the package is "solely dedicated to building, handling and plotting epidemic curves", although it also fits simple epidemic models to incidence data.

 Overall I think this package is sensible and well-designed. My only major concern is with the log-linear fitting procedures; on the one hand, the authors say clearly that there are many fitting procedures which cannot (and should not) all be squeezed into this package, and I suspect that they don't intend these fits to be taken as very precise measures of the growth rate. On the other hand, I'm quite concerned by the possibility that users will take growth rates and confidence intervals estimated by these simplistic fits too seriously. There are a number of delicate issues around the estimation of epidemic growth rates:

- Assume log-Normal incidence (as done by a log-linear fit), or make other distributional assumptions (e.g. Poisson or negative binomial)?
- If using exponential or log-linear fits, what time window should one use to capture enough of the beginning of the epidemic but not bias the growth rate downward by capturing the saturation phase of the epidemic (Ma *et al.*, 2014 ^1^)?
- Should one allow for the influence of both process and measurement error (King *et al.*, 2015 ^2^)?
- Is growth exponential or sub-exponential (Viboud *et al.*, 2016 ^3^)?

I think I would have preferred that, if they were not going to go deeply into this area, that the authors instead provide some sort of non-parametric smooth fit (perhaps constraining the changes to be monotonically increasing before the peak and decreasing after the peak) rather than oversimplifying in this way.

 The minimal change that is needed to the document is a stronger set of caveats/warnings to users that the log-linear model may have major shortcomings in some circumstances, and should not be accepted unquestioningly.

 The package suggests that the fit() function is extendable to allow other fitting methods ("While the incidence package returns incidence_fit objects containing log-linear models by default, they can be constructed from any model from which it’s possible to extract the growth rate (r) and predict incidence along the model"); I wasn't able to figure out how to achieve that goal.

 The main use of the package is for converting from line lists to aggregated incidence data. It would be useful (I can't tell if it's possible) to easily be able to aggregate data that are already in date/incidence form to coarser scales, or to approximately disaggregate incidence data.

 I had trouble fitting epidemic curves to data sets that only included incidence up to the peak or shortly beyond (it seems that the model was automatically trying to estimate a decline rate as well as an increase rate). This seems like an odd choice for a tool that people may be using to work with data from emerging epidemics that have not yet peaked.

**Minor comments:**

- p. 3, paragraph 1: missing space before (
- p. 3, paragraph 3: maybe R "language" or "environment" rather than "software" (R defines itself as "a language and environment for statistical computing and graphics")?
- Last paragraph of intro: comma before "which aims to fill this gap"?
- Methods, paragraph 2: Probably don't need to say "numeric and integer" values, "numeric" would suffice (is.numeric(1L) is TRUE in R).
- Methods, paragraph 2: "can not" -> "cannot".
- How do subsets and indexing work with dates?
- Figure 1 caption: delete "both"?
- "created from these where it checks and validates" -> "created from these components after checking and validating" ?
- "[Fitting] of log-linear models is depicted in the lower left"? (Technically, the addition of log-lin models to the plot is depicted along the bottom edge, or in the lower right).
- p. 5: "boo[t]strap".

I have read this submission. I believe that I have an appropriate level of expertise to confirm that it is of an acceptable scientific standard, however I have significant reservations, as outlined above.

## References

[1] MaJDushoffJBolkerBMEarnDJ: Estimating initial epidemic growth rates. *Bull Math Biol*.2014;76(1) : 10.1007/s11538-013-9918-2 245-60 10.1007/s11538-013-9918-2 24272389

[2] KingAADomenech de CellèsMMagpantayFMRohaniP: Avoidable errors in the modelling of outbreaks of emerging pathogens, with special reference to Ebola. *Proc Biol Sci*.2015;282(1806) : 10.1098/rspb.2015.0347 20150347 10.1098/rspb.2015.0347 25833863PMC4426634

[3] ViboudCSimonsenLChowellG: A generalized-growth model to characterize the early ascending phase of infectious disease outbreaks. *Epidemics*.15: 10.1016/j.epidem.2016.01.002 27-37 10.1016/j.epidem.2016.01.002 27266847PMC4903879
